# Supplementary material for: Divergence of cortical neurophysiology across different neurodegenerative disorders compared to healthy ageing
Source: Prog Neurobiol. Author manuscript; Available in PMC 2026 Mar 28. (PMC7618949; doi:10.1016/j.pneurobio.2025.102865)
Supplement: Supplementary Figure 1-4; Supplementary Table 1-2; Gray Matter Volume Extractor [file EMS212830-supplement-Supplementary_Figure_1_4__Supplementary_Table_1_2__Gray_Matter_Volume_Extractor.docx]

**Supplementary Material**
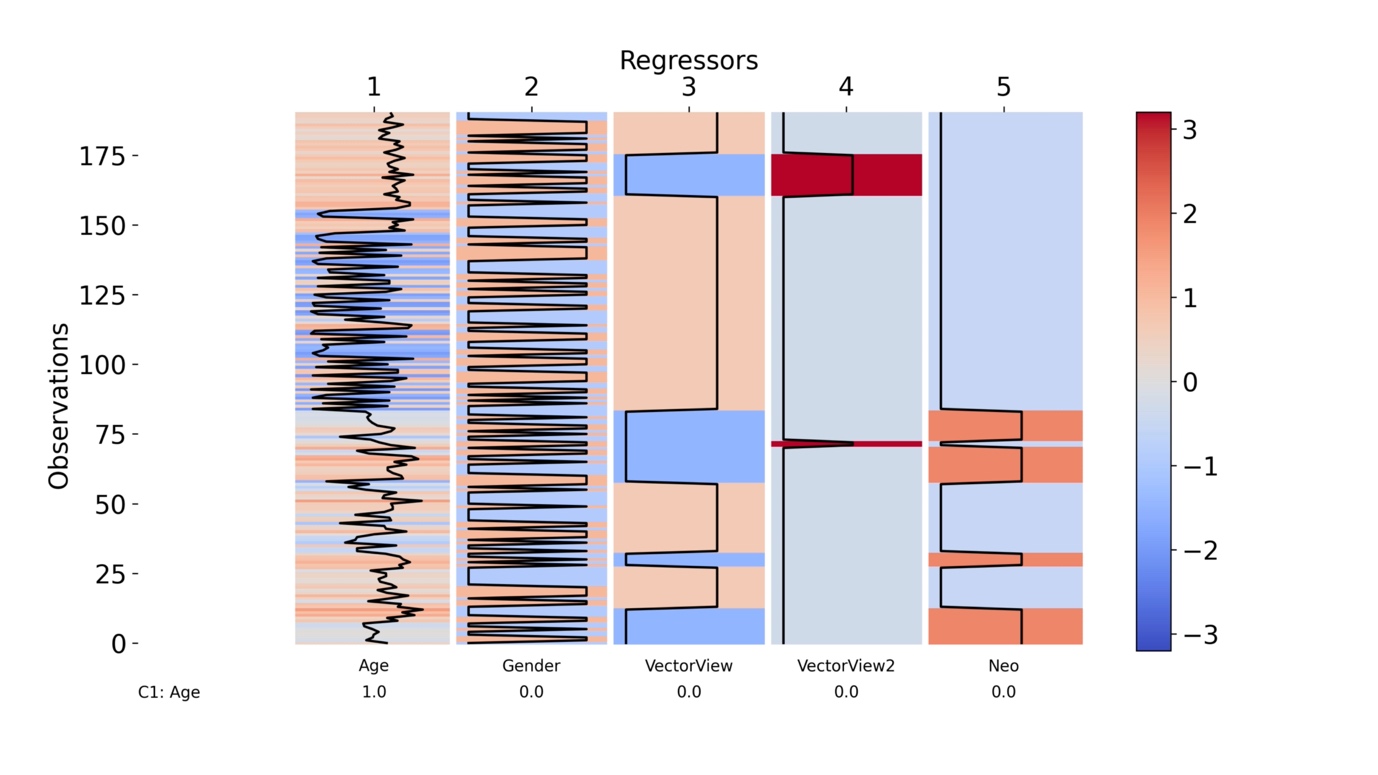


**Supplementary Figure 1 - General linear model design – healthy controls ageing**. Design matrix used to predict network metrics in healthy controls. The first regressor (age) models the z-transformed age values of the entire HC group (n = 191). The remaining regressors are included to model known sources of variability (sex, scanner type (VectorView, VectorView2, Neo)) across participants. This has the effect of minimising the impact of these confounds on the group means. The confound regressors are calculated by z-transforming the values for sex (1=female or 2=male) and scanner type (1 = scanner not used, 2 = scanner used). The last regressor models the mean across participants to allow comparison between groups.

**
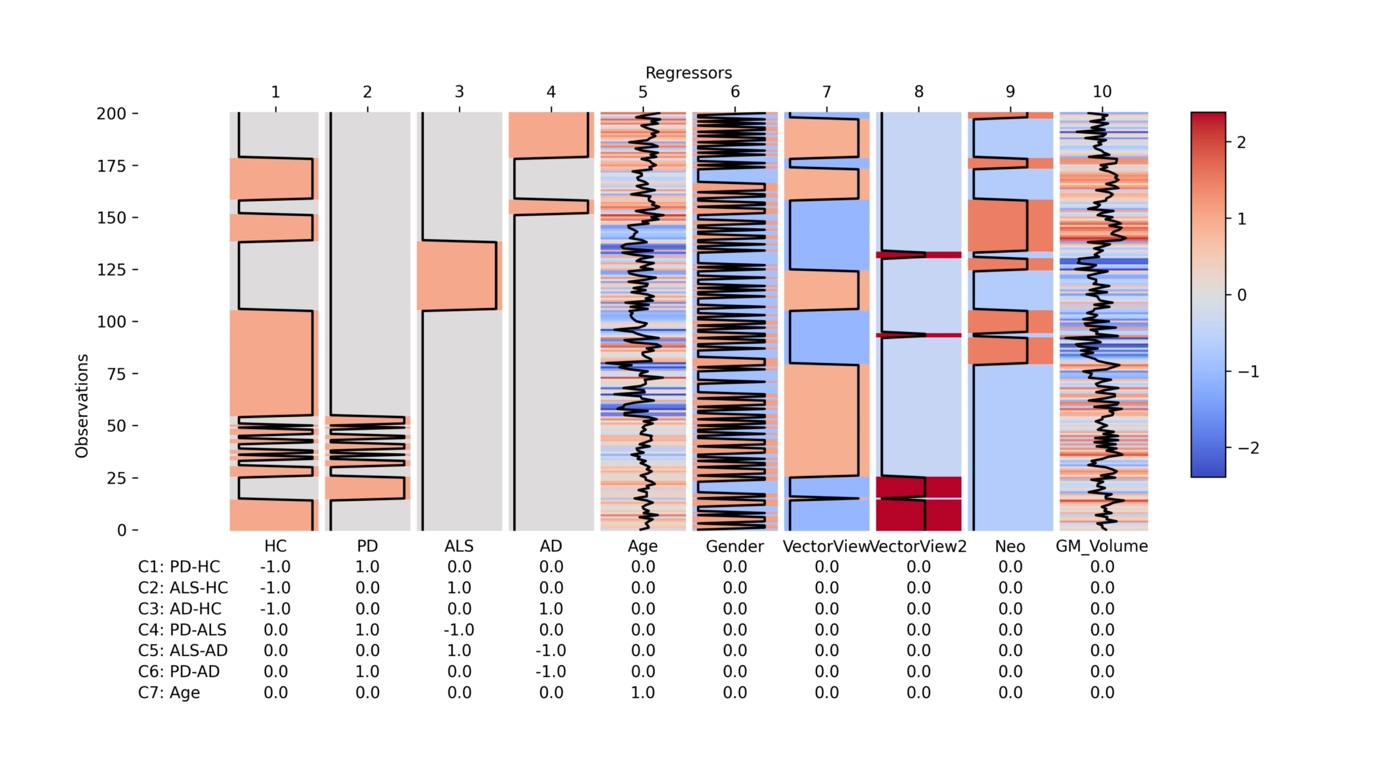
**

**Supplementary Figure 2 - General linear model design – group comparison**. Design matrix used to predict network metrics. The first regressor (HC) models the mean value of the network metric across healthy controls. The second, third and fourth regressors model the mean value of the network metric across PD, ALS and AD patients respectively. The remaining regressors are included to model known sources of variability (age, sex, scanner type (VectorView, VectorView2, Neo), grey matter volume (GM volume)) across participants. This has the effect of minimising the impact of these confounds on the group means. The confound regressors are calculated by z-transforming the values for age, sex (1=female or 2=male), scanner type (1 = scanner not used, 2 = scanner used), GM volume across participants.


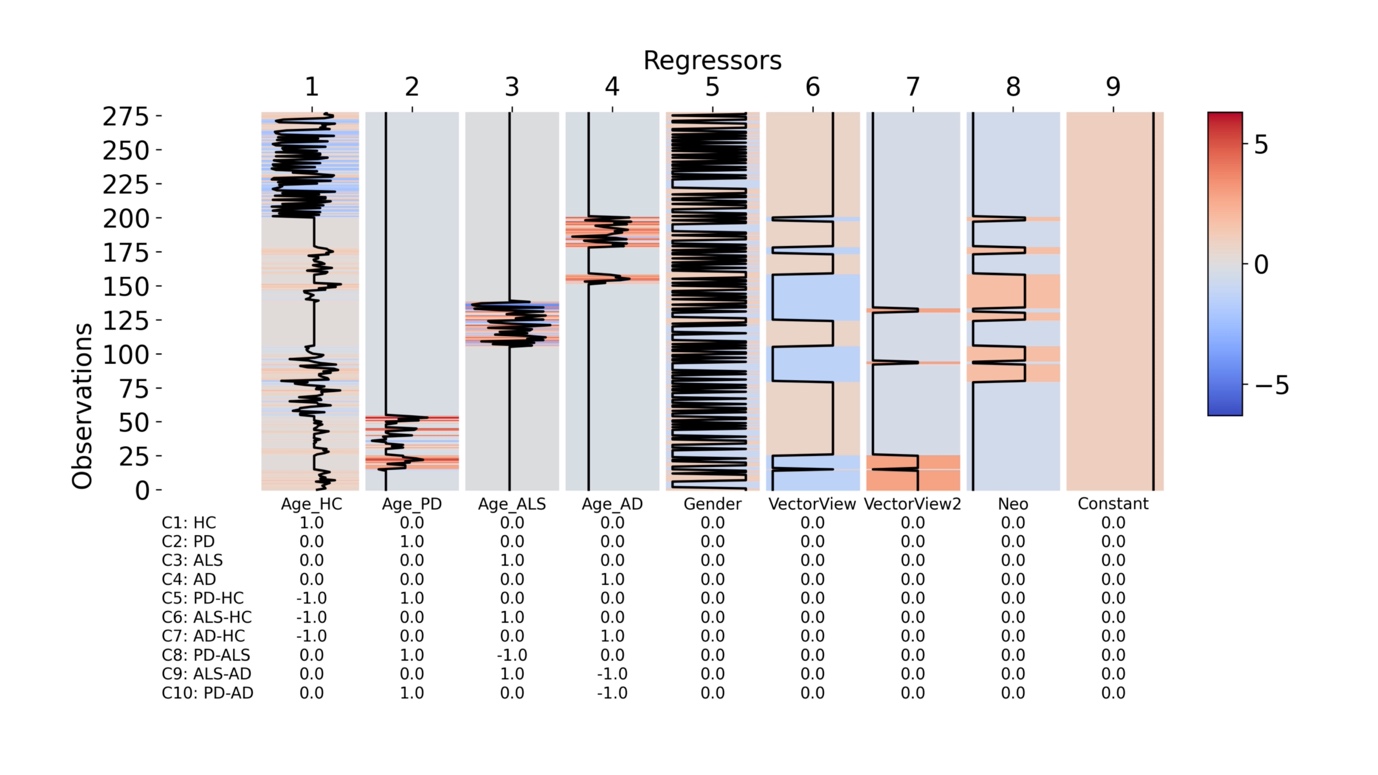


**Supplementary Figure 3 - General linear model design – group comparison**. Design matrix used to predict network metrics. The first regressor (HC) models the z-transformed ge values of the HC group. The second, third and fourth regressors model the z-transformed age values of the PD, ALS and AD patients respectively. The remaining regressors are included to model known sources of variability (sex, scanner type (VectorView, VectorView2, Neo)) across participants. This has the effect of minimising the impact of these confounds on the group means. The confound regressors are calculated by z-transforming the values for sex (1=female or 2=male) and scanner type (1 = scanner not used, 2 = scanner used). The last regressor models the mean across participants to allow comparison between groups.





**Supplementary Figure 4 - Beta power and age. A** - shows a scatterplot of the mean beta power values across all parcels. HC shows a significant increase in beta power with age (p <0.001). There was no significant effect of age on whole-brain beta power in PD, ALS or AD. **Bi** – shows the results of a GLM looking at the linear effect of age, parcel-wise within each group i.e. whether there was a significant linear relationship between age and beta power in each parcel. **Bii** – is comparing the linear effect of age on beta power between each disease group and HCs i.e. comparing line steepness in each parcel.

It was confirmed that the increase in beta was also seen in the older healthy group (N = 114) (r = 0.285, p = 0.008).





**Supplementary Figure 5 – Alpha and beta peak results. A** - shows changes associated with healthy ageing in a large group of HCs (N = 191). **B -** shows disease comparisons with age-matched healthy controls (AD, N = 29; PD, N = 25; ALS, N = 33 and age-matched HC, N = 114) whilst **C** - shows between-disease comparisons. Healthy ageing showed unchanged alpha and beta peak frequencies but significantly decrease alpha and beta peak power in occipital regions. Healthy ageing also showed increased alpha peak power in temporal regions and increased beta peak power in frontal, temporal and parietal regions. All neurodegenerative diseases showed a reduction in alpha peak frequency and beta peak power compared to controls. Compared to controls PD and ALS showed increased alpha peak power and decreased beta peak frequency whilst AD showed decreased alpha peak power and increased beta peak frequency. N.b. AD showed a reduced number of detectable beta peaks compared to controls (t(183) = -9.628, p < 0.001), ALS (t(183) = -7.682, p < 0.001) and PD (t(183) = -6.477, p < 0.001). There were no other significant differences in the number of detected alpha or beta peaks between groups (p > 0.1).

Supplementary Table 1 – Inclusion and exclusion criteria

|  | PD | ALS | AD |
| --- | --- | --- | --- |
| Inclusion  criteria | Participant is willing and able to give informed consent for participation in the study.  Participant is treated in neurological clinic in Oxfordshire (UK) or is included in Dementias and Neurodegeneration Speciality (<https://dendron.org.uk>) database. | For all participants:  Age≥18  Participant is willing and able to give informed consent for participation in the study, or an appropriate consultee can be identified to provide advice about his/her friend or relative’s inclusion in the study. If the participant is unable to provide written consent due to physical disability, an independent witness will be present at the informed consent discussion and sign the consent form on the participant’s behalf.  Additional criterion for patients with ALS:  Diagnosis made by experienced neurologist | Patients will have a diagnosis of early Alzheimer’s disease (MMSE>18, CDR=0.5-1). For healthy controls the score cut-offs are MMSE>24 and CDR=0.  Men and women aged 50 years and above with an upper age limit of 85, at baseline visit.  Participants must understand the nature of the study and must provide signed and dated written informed consent prior to initiation of any study-related procedures. Participants who are deemed incapable of providing informed consent may not be enrolled.  Patients will show evidence of cognitive impairment as measured on cognitive tests, in accordance with study recruitment requirements.  Cerebral amyloid status as determined either by PET or CSF sampling, in accordance with study recruitment requirements.  Rosen Modified Hachinski Ischaemic score of ≤ 4.  The participant should be mentally and physically able to understand and participate in all scheduled assessments and to complete all required tests and procedures, as judged by the Investigator, including PET imaging, lumbar punctures and computerised assessments.  Able to read and write and with minimum 7 years of formal education (full time school college, university, but not including vocational training schemes and manual apprenticeships)  The participant must be healthy, as determined by the Investigator, based on medical evaluation including medical history, physical examination and laboratory tests, as appropriate.  The participant must be able to lie comfortably on their back for up to 90 minutes at a time.  The participant must be able to walk independently for at least 10 metres, without a walking aid. |
| Exclusion  criteria | Zokaei et al. (2021):  Currently not participation in ongoing drug trial  Not taking any of the following drugs:  Psychotropic hypertensive  Vasoactive medication  Long-acting dopamine agonists  No history of neurological or psychiatry disorders other than PD.  Tolerating coming off dopaminergic medication.  Heideman et al. (2020):  Being diagnosed with PD within the last 5 years.  Being able to understand written and spoken instructions in English.  Being older than 50 years.  Tolerating coming off dopaminergic medication. | Age <18 years  Pregnancy or any other condition that in the opinion of the investigator could interfere with the interpretation of the study results.  • Significant bleeding diathesis or sepsis, or other condition that in the opinion of the investigator could constitute a health risk to the participant if he/she took part in this element of the study.  Significant orthopnea or secretions, defined as subjectively intolerable breathlessness on lying flat or oxygen saturations below 90% on lying flat for 10 minutes.  Severe physical disability that might preclude accessing the scanner or prevent communication of any distress during testing or presence of any incompatible (or possibly incompatible) metal implants or large tattoos suspected to contain iron pigments.  • An implantable cardioverter-defibrillator (ICD) device. | Significant neurological disease affecting the CNS, other than early AD, that may affect cognition or ability to complete the study, including, but not limited to: other dementias, serious infection of the brain, epilepsy or Parkinson’s disease.  Any clinically important abnormality as determined by the Investigator at screening or between screening and Day 1, in medical history, physical examination, neurological examination (including significant focal findings), vital signs or clinical laboratory test results that could be detrimental to the participant or could compromise the study.  Presence of any significant psychiatric disorder, according to the criteria of the Diagnostic and Statistical Manual of Mental Disorders, 5th Edition – Text Revision (DSM-V), including but not limited to addictive disorders, or symptom, if, in the judgement of the Investigator, the psychiatric disorder or symptom is likely to confound interpretation of cognitive assessment or affect the participant’s ability to complete the study. Participants with mild affective disorder may be included if, in the judgment of the investigator, the disorder is not sufficient to explain the cognitive deficits.  Medications with the potential to significantly affect cognition, including but not limited to: anxiolytics, antidepressants, antipsychotics and sedative-hypnotics, are not permitted unless maintained on a stable dose regimen for at least 30 days prior to the baseline visit.  Impairment of vision or hearing that would make cognitive testing, MEG or EEG testing difficult, as judged by the Investigator. Neither hearing aids nor standard glasses can be worn in the MEG system. Hearing frequencies at certain frequencies may be tested using a brief auditory threshold paradigm. Each MEG centre will inform the Investigator of the range of optical prescriptions that can be satisfactorily corrected using MEG-compatible glasses.  History of multiple episodes of minor head trauma or any head trauma resulting in protracted loss of consciousness, or serious infectious disease affecting the brain, within five years of screening or between screening and baseline visit.  History of seizure, except febrile seizures or single provoked seizure.  A clinically significant illness (including chronic, persistent or acute infection), medical or surgical procedure, or trauma within 30 days prior to screening or between screening and baseline visit. |

**Supplementary Table 2:** Glasser52 parcellation co-ordinates. Parcel names and MNE-coordinates of parcel centres of the Glasser52 Parcellation.

| Region Index | Location description | X | Y | Z |
| --- | --- | --- | --- | --- |
| 0 | Primary and Early Visual Cortex Right | 14.5 | -80.5 | -0.4 |
| 1 | Dorsal Stream Visual Cortex Right | 19.4 | -81.4 | 30.6 |
| 2 | Ventral Stream Visual Cortex Right | 29.9 | -57.6 | -17.7 |
| 3 | MT+ Complex and Neighbouring Visual Areas Right | 42.2 | -71.5 | 0.0 |
| 4 | Superior Somatosensory and Motor Cortex Right | 23.6 | -29.5 | 61.0 |
| 5 | Inferior Somatosensory and Motor Cortex Right | 49.1 | -13.4 | 38.3 |
| 6 | Supplementary Motor Area Right | 12.7 | -2.2 | 62.9 |
| 7 | Cingulate Motor Areas & Area 5 Right | 10.0 | -31.2 | 53.6 |
| 8 | Premotor Cortex Right | 40.9 | 0.9 | 41.1 |
| 9 | Insular & Frontoparietal Operculum Right | 37.7 | 1.6 | 4.0 |
| 10 | Early Auditory Cortex Right | 40.2 | -27.5 | 13.5 |
| 11 | Auditory Association Cortex Right | 55.0 | -14.0 | -6.5 |
| 12 | Medial Temporal Cortex Right | 25.0 | -22.1 | -22.1 |
| 13 | Lateral Temporal Cortex Right | 48.5 | -16.3 | -25.3 |
| 14 | Temporal-Parieto-Occipital Junction Right | 52.1 | -48.1 | 12.3 |
| 15 | Medial Bank of the Intra-parietal Sulcus Right | 29.5 | -51.0 | 43.0 |
| 16 | Superior Medial Parietal Cortex Right | 19.1 | -58.4 | 59.6 |
| 17 | Inferior Parietal Cortex Task-Positive Network Right | 56.7 | -29.7 | 35.7 |
| 18 | Inferior Parietal Cortex Task-Negative Network Right | 47.4 | -56.1 | 35.8 |
| 19 | Intraparietal Sulcus & PGP Right | 36.5 | -71.1 | 32.6 |
| 20 | Posterior Cingulate Cortex Right | 10.3 | -55.1 | 26.8 |
| 21 | Anterior Cingulate and Medial Prefrontal Cortex Right | 6.1 | 33.8 | 14.2 |
| 22 | Orbital and Polar Frontal Cortex Right | 17.1 | 47.0 | -11.6 |
| 23 | Inferior Frontal Cortex Right | 45.4 | 32.5 | 3.8 |
| 24 | Inferior Dorsolateral Prefrontal Cortex Right | 34.8 | 37.1 | 25.7 |
| 25 | Superior Dorsolateral Prefrontal Cortex Right | 21.0 | 31.9 | 45.9 |
| 26 | Primary and Early Visual Cortex Left | -16.9 | -82.0 | -0.9 |
| 27 | Dorsal Stream Visual Cortex Left | -21.7 | -84.3 | 27.0 |
| 28 | Ventral Stream Visual Cortex Left | -34.5 | -56.0 | -18.6 |
| 29 | MT+ Complex and Neighbouring Visual Areas Left | -45.2 | -70.3 | -1.4 |
| 30 | Superior Somatosensory and Motor Cortex Left | -22.6 | -30.4 | 62.8 |
| 31 | Inferior Somatosensory and Motor Cortex Left | -48.3 | -18.7 | 41.7 |
| 32 | Supplementary Motor Area Left | -14.0 | -2.7 | 62.7 |
| 33 | Cingulate Motor Areas & Area 5 Left | -14.4 | -30.6 | 49.7 |
| 34 | Premotor Cortex Left | -40.3 | -1.7 | 43.8 |
| 35 | Insular & Frontoparietal Operculum Left | -41.3 | 0.1 | 3.7 |
| 36 | Early Auditory Cortex Left | -45.4 | -29.4 | 12.1 |
| 37 | Auditory Association Cortex Left | -55.3 | -17.3 | -7.8 |
| 38 | Medial Temporal Cortex Left | -27.9 | -22.2 | -22.6 |
| 39 | Lateral Temporal Cortex Left | -49.6 | -17.9 | -25.1 |
| 40 | Temporal-Parieto-Occipital Junction Left | -53.1 | -54.0 | 14.5 |
| 41 | Medial Bank of the Intra-parietal Sulcus Left | -31.1 | -50.0 | 41.1 |
| 42 | Superior Medial Parietal Cortex Left | -21.3 | -60.5 | 58.2 |
| 43 | Inferior Parietal Cortex Task-Positive Network Left | -56.6 | -36.3 | 36.6 |
| 44 | Inferior Parietal Cortex Task-Negative Network Left | -45.1 | -63.4 | 35.2 |
| 45 | Intraparietal Sulcus & PGP Left | -36.7 | -70.0 | 30.4 |
| 46 | Posterior Cingulate Cortex Left | -11.6 | -49.5 | 28.0 |
| 47 | Anterior Cingulate and Medial Prefrontal Cortex Left | -8.7 | 32.0 | 11.4 |
| 48 | Orbital and Polar Frontal Cortex Left | -21.9 | 46.3 | -8.1 |
| 49 | Inferior Frontal Cortex Left | -45.1 | 28.2 | 7.7 |
| 50 | Inferior Dorsolateral Prefrontal Cortex Left | -37.2 | 36.2 | 25.2 |
| 51 | Superior Dorsolateral Prefrontal Cortex Left | -23.5 | 28.6 | 45.7 |

**Grey Matter Volume Extraction**

Volumetric analysis of cortical and subcortical structures was completed using FSL v6.0.7.3 (Smith et al., 2004; Woolrich et al., 2009). The FSL Anatomical Preprocessing pipeline (FSL anat) was used for reorientation to standard (MNI) space, automatic cropping, bias field correction, registration to standard space, brain extraction, tissue-type segmentation and subcortical structure segmentation according to the Harvard-Oxford subcortical segmentation atlas (Jenkinson et al., 2012; Smith et al., 2004; Woolrich et al., 2009). Total white matter, grey matter and CSF volumes were extracted. Normalised grey matter volume was calculated as a proportion of total intracerebral volume.

**References**

Jenkinson, M., Beckmann, C.F., Behrens, T.E.J., Woolrich, M.W., Smith, S.M., 2012. FSL. NeuroImage, 20 YEARS OF fMRI 62, 782–790. https://doi.org/10.1016/j.neuroimage.2011.09.015

Smith, S.M., Jenkinson, M., Woolrich, M.W., Beckmann, C.F., Behrens, T.E.J., Johansen-Berg, H., Bannister, P.R., De Luca, M., Drobnjak, I., Flitney, D.E., Niazy, R.K., Saunders, J., Vickers, J., Zhang, Y., De Stefano, N., Brady, J.M., Matthews, P.M., 2004. Advances in functional and structural MR image analysis and implementation as FSL. Neuroimage 23 Suppl 1, S208-219. https://doi.org/10.1016/j.neuroimage.2004.07.051

Woolrich, M.W., Jbabdi, S., Patenaude, B., Chappell, M., Makni, S., Behrens, T., Beckmann, C., Jenkinson, M., Smith, S.M., 2009. Bayesian analysis of neuroimaging data in FSL. Neuroimage 45, S173-186. https://doi.org/10.1016/j.neuroimage.2008.10.055
